# ECG Response of Koalas to Tourists Proximity: A Preliminary Study

**DOI:** 10.1371/journal.pone.0007378

**Published:** 2009-10-12

**Authors:** Yan Ropert-Coudert, Lisa Brooks, Maki Yamamoto, Akiko Kato

**Affiliations:** 1 Institut Pluridisciplinaire Hubert Curien, UMR7178, Strasbourg, France; 2 Koala Conservation Centre, Phillip Island Nature Park, Cowes, Victoria, Australia; 3 Department of Bioengineering, Nagaoka University of Technology 1603-1, Kamitomioka, Nagaoka, Niigata, Japan; 4 National Institute of Polar Research, Itabashi-ku, Japan; Pennington Biomedical Research Center, United States of America

## Abstract

Koalas operate on a tight energy budget and, thus, may not always display behavioral avoidance reaction when placed in a stressful condition. We investigated the physiological response of captive koalas *Phascolarctos cinereus* in a conservation centre to the presence of tourists walking through their habitat. We compared, using animal-attached data-recorders, the electrocardiogram activity of female koalas in contact with tourists and in a human-free area. One of the koalas in the tourist zone presented elevated heart rate values and variability throughout the recording period. The remaining female in the exhibit area showed a higher field resting heart rates during the daytime than that in the isolated area. In the evening, heart rate profiles changed drastically and both the koalas in the exhibit and in the tourist-free zones displayed similar field resting heart rates, which were lower than those during the day. In parallel, the autonomic nervous systems of these two individuals evolved from sympathetic-dominant during the day to parasympathetic-dominant in the evening. Our results report ECG of free-living koalas for the first time. Although they are preliminary due to the difficulty of having sufficient samples of animals of the same sex and age, our results stress out the importance of studies investigating the physiological reaction of animals to tourists.

## Introduction

The koala *Phascolarctos cinereus*, once present over a large area of the Australian continent, is now listed by the IUCN as a vulnerable species (even considered rare in New South Wales and South Australia) and its distribution range is restricted to the east coast of Australia [1]. Causes of mortality are diverse, although disease (essentially chlamydiosis), collisions with cars and loss of habitat represent the principal reasons for the species continuous decrease. Among various conservation approaches, conservation centers have been created that aim at simultaneously protecting the koala habitat, and instructing the public. In these sanctuaries, the natural habitat of the koala is re-created and, in some instances, boardwalks crossing the canopy of the Eucalyptus forests used by the koalas, allow tourists to view animals from a close range. Captive koalas seem unaffected when approached by tourists as they show no exterior signs of embarrassment, stress or disturbance, do not try to escape or, if they move out from the tourists, they do it as a slow pace.

However, animals in stressful situations do not always show visible reactions. Escape is only one of the possible responses but some animals adopt other strategies, such as staying motionless while mimicking the environment. In some instance, stress can instead be revealed through an abnormally elevated heart rate in those species that remain motionless when approached by human. Hence, Adélie penguins *Pygoscelis adeliae* incubating on their nests display resting heart rates of 86 beat per minute (bpm) but this rises to 127 bpm when approached by visitors [2]. Similarly, giant petrels *Macronectes hallii* display an elevated cardiac response to approaching tourist while sitting motionless on their nests [3]. Classically, an increase in heart rate corresponds to an increase in metabolic rate. Although the relationship between stress-related heart rate and metabolic rate is less clear [4]–[6], [7] showed that these two factors increase concomitantly but metabolic rate remains elevated for a while after the heart rate has returned to its original value. Heart rate appears thus as a relevant measure of perceived stress in motionless animals.

In this regard, koalas can be expected to display limited stress-response, at least visually, because of their highly-specialized feeding habits. Their diet consists exclusively of Eucalyptus leaves that are extremely poor in proteins and also contains secondary metabolites (e.g. terpens, lignins...) that need to be detoxified before being assimilated [8]. In other words, koalas ingest little amount of energy and expend a part of it to eliminate the toxic compounds of their food. Consequently, they have evolved a series of adaptations to save up energy, e.g. reduced activity (ca. 4 h per day, during the early evening), specific, physiological adaptations of their digestive systems, etc. [1]. With such a tight regime, the absence of escape behavior of koala due to a stressful situation could be regarded as an energy-saving behavior [1]. Thus, if the proximity of tourists contributes to raise their heart rates, there is a risk that this would lead to an extra-cost in the energy expanded, a currency that they may not be able to afford on the long term.

Heart rates of freely-living koalas have never been reported in the literature. The purpose of the experiment was to investigate, for the first time, the physiological response (heart rate and autonomic nervous system) of koalas to visitors approaching them from a boardwalk. One inevitable constraint of such a protocol when dealing with koalas is the rarity of settings where we could find enough animals to form these two categories, especially since individuals should be of the same age, sex and monitored on the same day. There are also numerous ethical and logistical restrictions that prevent from obtaining large sample size. Yet, it seems more valuable to us to conduct an experiment on a limited sample rather than to have no information whatsoever on the impact of human approach onto this sensitive species. Using the least possible invasive method, we were able to compare the heart rates of two koalas in an exhibit area with daily exposure to tourists, with that of a koala kept in similar semi-captive conditions but in a tourist-free area. We hypothesize that the koalas exposed to tourists would display higher resting (i.e. during periods of inactivity) heart rates than the tourist-free koala.

## Materials and Methods

### Study site and Animals studied

The Koala Conservation Centre is located on Phillip Island (38°29′S, 145°15′E), Victoria, Australia, 140 km south-east of Melbourne. It is part of the Phillip Island Nature Park and opened to visitors from 10 AM to 5 PM.

We compared two koalas that were in two separate boardwalk areas, *Nugget* and *Crimson*, and one koala kept in a 15×15 m pen in the tourist-free area, *Nemo*. All were females (to avoid a potential sex bias), and of similar ages (2 individuals were 2-yrs old and one 5 years old), without joeys and non pregnant (Table 1). They were weighed before and after instrumentation to the nearest 10 g, using a spring balance.

**Table 1:** Summary of freely-living koalas used at the Koala Conservation Center, Phillip Island, Australia. Heart rate values are given as means ± 1SD, and the range is indicated in parentheses.

	Nemo	Crimson	Nugget
Sex	F	F	F
Age (yrs)	2	2	5
Mass (kg)	7.2	6.0	8.1
Location	Holding pen	Boardwalk	Boardwalk
Number of visitors	0	110	337
Mean distance to visitors (in m)	0	ca. 7–8	ca. 1–3
Heart rate during inactivity (1–6PM) in bpm	105.3±5.4 (82.6–126.0)	111.1±10.0 (81.3–134.5)	113.8±4.1 (90.6–129.3)
Heart rate during inactivity (6–10PM) in bpm	94.6±10.6 (56.4–114.5)	120.7±8.8 (90.1–142.9)	97.7±10.3 (56.2–120.5)
ODBA during the day (2–5 PM) (G)	0.0069	0.0137	0.0108
ODBA during the night (8–10 PM) (G)	0.0143	0.0159	0.0113

### Ethics Statement

This study was done under permits 1/2007 from the Phillip Island Nature Park Animal Experimentation Ethics Committee, Phillip Island, Victoria, Australia. We followed the animal husbandry guidelines in use at the Koala Conservation Center in Phillip Island. Capture and handling were performed by trained rangers of the Park.

### Data-loggers

We used a miniaturized, cylindrical electrocardiogram recorder sampling at 500 Hz (UWE-380-ECG, 12 bit resolution, 105×20 mm, 52 g, Little Leonardo). This system was attached externally, hence requiring no surgery and no anesthesia [9]. It is composed of a cylindrical body from which three 5–7 cm cables (the electrodes) emerged. The cables end in plating (to avoid infection) safety pins that serve as electrodes. The pins were stitched to an approx. 1 cm diameter patch of bare skin cleansed with 70° Ethanol, on the back of the koala. The logger recorded the voltage between 2 electrodes at a range of −5.9 to +5.9 mV, with 2.88×10^−3^ mV resolution in an 8 MB flash memory. The reliability of the data recorded by this system was previously tested on Adélie penguins, great cormorants *Phalacrocorax carbo hanedae* and more recently on Cape gannets *Morus bassanus*. We also checked the reliability of the signal recorded by the logger using an ECG monitor (HeartMate, IEC-1103, Nihon-Koden) on a hand-held koala before the start of the experiment. The pulses recorded by the monitor and the logger were identical. The high sampling frequency also meant that the memory became full after half a day approximately, so all ECG recorders were programmed to start sampling 45 hours after the attachment on the koala. Such a delay ensured that the heart rates of the koalas return to a normal level before the start of the experiment.

In parallel, a miniature, cylindrical, data logger (M190-D2GT, 12 bit resolution, 53×15 mm, 17 g, Little Leonardo) was attached along the body of the ECG recorder. This data-loggers simultaneously monitored acceleration along 2 axes (sampling frequency: 16 Hz on each axis): surging along the longitudinal axis of the koala and heaving along the dorso-ventral axis [10]. Accelerometers were used to monitor continuously the koala activity, even when those were not under visual surveillance, so as to determine if animals were moving or not.

Both loggers were temporarily attached to the fur of the upper back of the koalas (between the shoulder blades) using adhesive tape (Tesa, Germany) allowing for a quick attachment and retrieval of the devices (without causing damage to the fur). They were all set to start recording on the same day to ensure that all koalas experienced similar weather conditions.

Koalas were captured using a noose at the end of a pole. The noose was placed and tightened around the neck of the koala to prevent it from climbing higher. A red flag was flashed above the head of the animal to force it to descend the tree. Once on the ground, koalas were kept in a capture bag, with only the top of their back free. Data-loggers were attached according to the method described above, the whole process lasting about 20 min. Recapture for logger removal occurred about a week later when all koalas in the Center were caught at the occasion of the annual health check. No marks of extensive grooming (fur damaged, bare skin patch) or infection/irritation were observed at the point of insertion of the electrodes or at the logger attachment sites. The point of needle insertion was cleansed again with Ethanol.

### Observation sessions

Nugget and Crimson were observed during the day when the ECG loggers were recording. The observation period was divided into “day” (from 1PM to 6 PM) and “evening” (from 6 PM to the end of the recording, i.e. 10 PM), which correspond to mainly inactive and mainly active periods, respectively. Koalas were observed with the naked eye and binoculars, from approx. 15 m distance to avoid the risk of interfering with the experiment. During the observation period, from 1 PM to 8 PM, three pauses occurred (one long from 3:20 PM to 4:00 PM, and two 5–10 min pauses around 5:30 PM and 6:20 PM). Nugget was observed from the boardwalk and Crimson from outside the boardwalk. Observers noted simultaneously the activity of the koalas (sleeping, scratching, awake but not moving, feeding, shifting position and climbing), as well as the time and number of people passing within a horizontal 5-m radius of the koala (both koalas were in trees, ca. 10 m high and 2–3 m high from the tourists for Crimson and Nugget, respectively). As part of the study, an experimenter walked around the trees of Nugget (around 7:00 PM) and Crimson (around 6:45 PM) after all visitors had departed.

### Analysis

**Figure 1:**
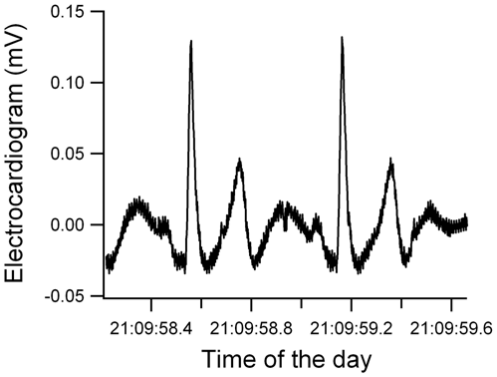
An excerpt of the electrocardiogram signal of Nugget showing the PQRT peaks.

Following recovery, data were downloaded onto a computer and analyzed using Igor pro (Wavemetrics v. 6.0.4.0). We first used a purpose-written macro to identify each R peak individually (Fig. 1). All sections where ECG peaks could not be clearly identified because of the noise from muscles were manually removed from the analysis. We then calculated the immediate heart rate from the R-R interval (RRi in sec) as:

Heart rate (in beat per minute, bpm)  = 60/RRi

Using Ethographer v.1.3 [11], the RRi were interpolated with a linearly spline and then re-sampled at every 100 ms and a Fast Fourrier Transform was conducted on the re-sampled data over periods comprising 2048 data points with a step of 5 sec. Power spectral analysis of heart rate variability is an indirect estimation of the cardiac autonomic tone [12]–[14]. In mammals, a power spectral analysis of heart rate variability generates peaks at generally two frequency bands (occasionally a third band, a very low frequency band is also recognized), a low frequency (LF) and a high frequency (HF) bands [14]–[15]. The range of the HF peak generally reflects the respiratory rate. As koalas have been reported to have a respiratory rate around 10–15 breaths/min [16], we can expect a HF band around 0.15–0.25 Hz, like for horses, which present a similar respiratory rate [14]. In addition, we also calculated two other parameters indicative of heart rate variability: the SDNN, i.e. the standard deviation of all R-R intervals in the dataset; and the RMSSD, i.e. the square root of the mean of the sum of the squares of the differences between successive R-R intervals (in other words, the standard deviation of the differences of adjacent R-R intervals).

From the power spectral analysis, the area under the curve of the low frequency peak is considered to reflect the combination of the cardiac sympathetic and parasympathetic activities, whereas that under the high frequency band indicates parasympathetic activity. Similarly, SDNN and RMSSD are affected by both sympa- and parasympathetic nervous activities and only parasympathetic activity, respectively. In this regard, the ratio (SDNN/RMSSD) can be used as an index of the balance between those two nervous activities (similar to the LF/HF ratio).

We finally calculated an index of activity, the Overall Dynamic Body Acceleration (ODBA, see [17] for details of the calculation), using the acceleration values for day (average between 2–6 PM) and night (6–10 PM). This allowed us to identify periods of inactivity, on which we applied the power spectral analysis.

We used a two-way Anova to test differences in heart rates between koalas and between periods of the day, followed by Student t post-hoc tests. The statistical threshold was 0.05. Statistical tests were conducted using JMP (SAS Institute Inc., USA, version 5.1.1J).

## Results

The weather on the observation day (Fri 9 Nov 2007) was fine (16°C on average, 20°C max, Australian Meteorological Bureau) with little wind (11 km/h on average). We obtained 9.68 h, 9.65 h, and 9.73 h of ECG recordings for Nugget, Crimson, and Nemo, respectively. In parallel, we recorded 75.13 h, 75.1 h, and 107.22 h of activity on Nugget, Crimson and Nemo, respectively.

### Activity and Reactivity

During the day (1PM–5PM) approx. a 110 people passed near the tree where Crimson stayed 8–10 m above the boardwalk. Couples were the most frequent and the noise level was mainly normal with a few medium levels when larger groups stayed in the boardwalk. Crimson neither showed any visible reaction nor did she move from her position during that time. In contrast, Nugget occasionally moved to and from her favorite feed station on the ramp of the boardwalk, i.e. about 1 m from the tourists, staying 2–3 m above the boardwalk when not feeding. Nugget was visited by 337 tourists during the day and was slightly more active than Crimson, as she fed once during the afternoon and again in the evening. Overall, most of the day activity for the three koalas, determined visually, consisted in scratching, shifting position, and sleeping.

Accelerometers confirmed that all koalas were more active during the night than the day (Table 1). The day-night differences were the largest for Nemo and the smallest for Nugget.

### Physiological reaction

**Figure 2:**
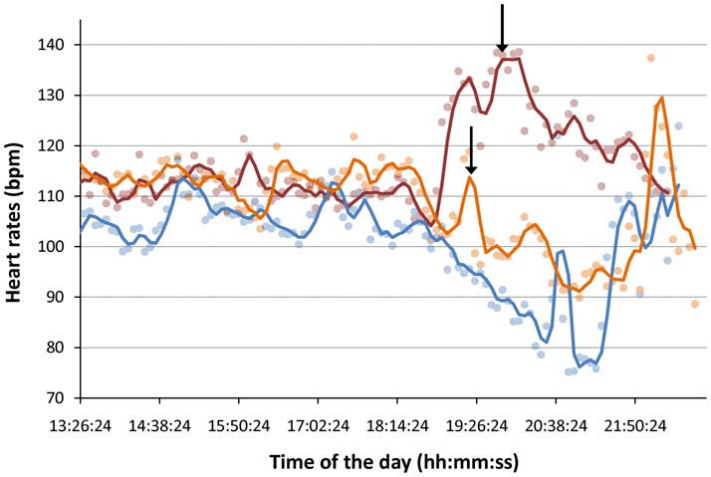
Heart rates of Crimson (red), Nugget (orange) and Nemo (blue), averaged every 5 minutes (dots) over the whole recording periods. A curve depicting the moving average of the heart rate, calculated with a 3 points step is superimposed on the dots. Note the drastic change in heart rate around 6:30 PM for all koalas. Arrows indicate the time at which an experimenter walked around the trees of Crimson and Nugget.

The heart rates during the day (1–6 PM) remain relatively constant and did not vary much in comparison to the evening (Table 1). The transition between the day and evening was sharp and occurred in a remarkably synchronized manner between the three koalas (around 6:30 PM, Fig. 2). Both Nugget and Nemo showed similar overall decreasing trends in the evening (starting at 6:30 PM and 6:37 PM, respectively). In contrast, Crimson's heart rate increased at first (from 6:27 PM) before decreasing after 8PM. During the evening period, transitory peaks of tachycardia were frequently observed, some of them coinciding with the experimenter walking under the trees of Nugget and Crimson and cracking branches (arrows on figure 2) and also with bouts of activity in the three koalas, e.g. eating or climbing.

**Figure 3:**
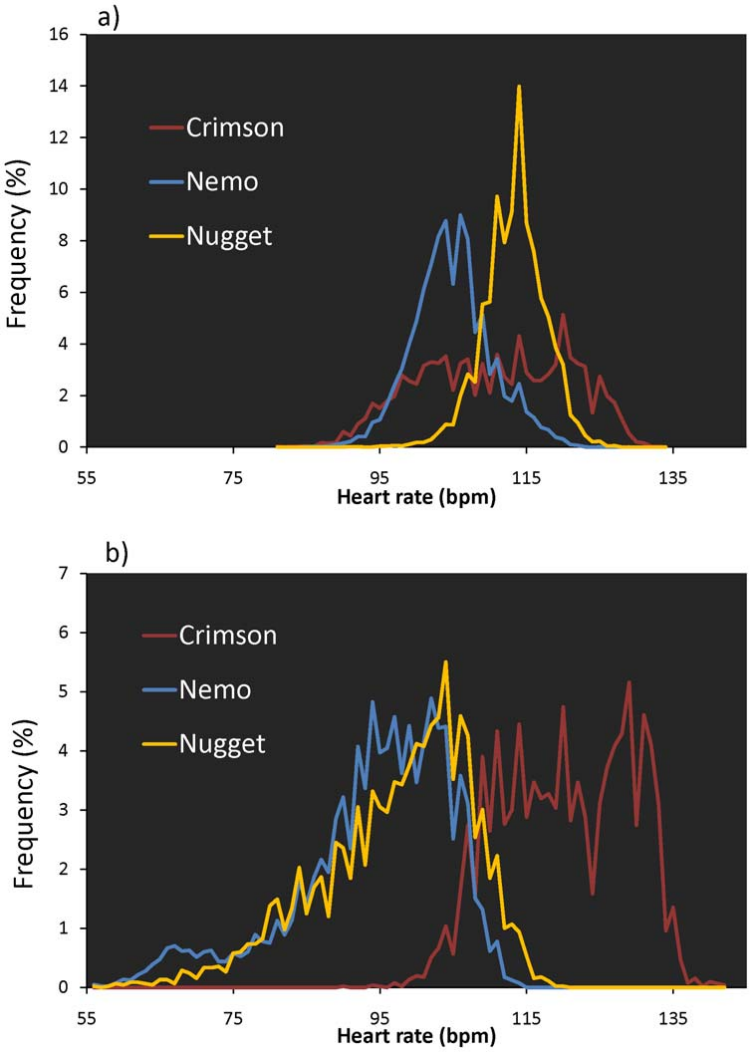
Heart rates recorded during periods of complete inactivity over a) the day (1:00–6:00 PM) and b) the evening (6:00–10:00 PM) of the experiment for the two koalas in contact with tourists (Nugget and Crimson) and the koala without human contact (Nemo).

To compare the heart rates of koalas in presence and absence of tourists, we only considered periods of complete inactivity, as determined from visual estimation and/or accelerometer signals. The heart rates, hereafter, referred to as field resting heart rates (HR), of the three koalas differed significantly among individuals and between the day (before 6 PM) and the night (after 8 PM) periods (F_5,44_ = 44.42, P<0.0001). During the day, Nemo displayed a lower HR than the two other koalas, whose HR did not differ (Fig. 3). In the evening, only the HR of Crimson was significantly greater than that of the two other koalas, whose HR were not significantly different. In addition, the HR of Crimson was highly variable during the day (coefficient of variation C_v_ = 0.09, i.e. about twice the C_v_ of Nemo and Nugget, 0.05 and 0.04, respectively). This trend reversed during the evening periods, where Nemo and Nugget had similar C_v_ (0.11) that were higher than that of Crimson (0.07).

**Figure 4:**
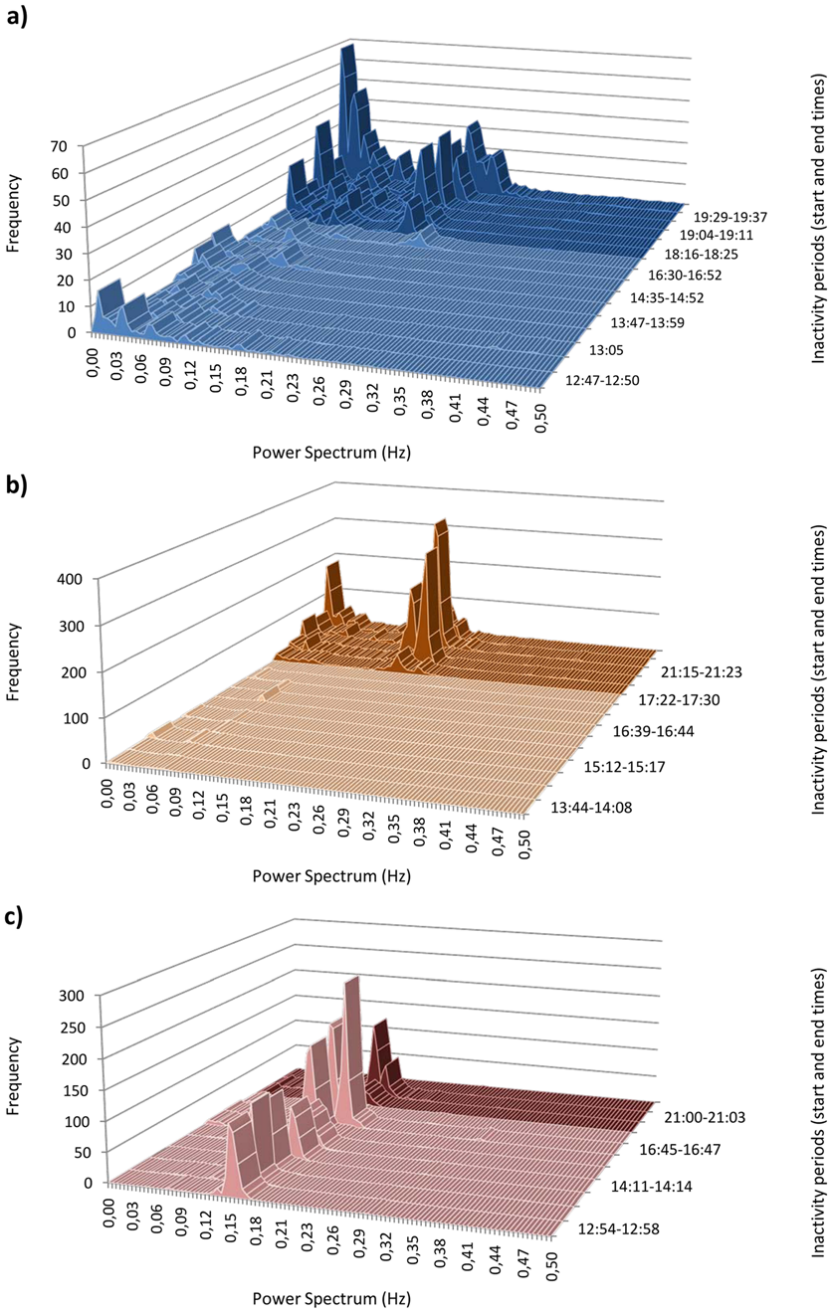
Spectral analysis of the heart rate variability during periods of inactivity throughout the recording periods for a) Nemo (no contact with human), b) Nugget and c) Crimson.

**Figure 5:**
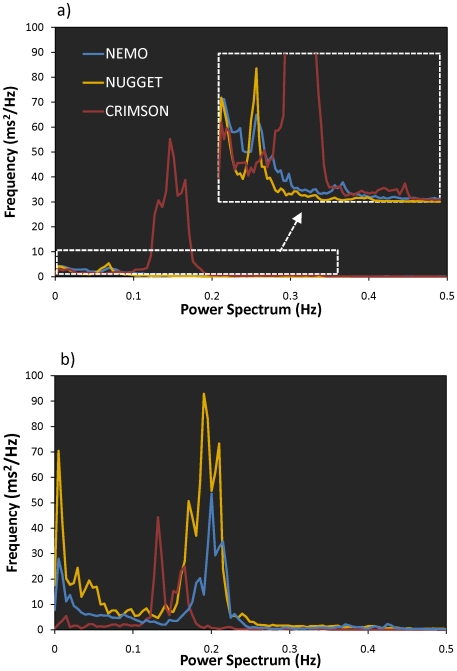
Averaged spectral analysis of the heart rate variability over the periods of inactivity of the two koalas in contact with tourists (Nugget and Crimson) and the koala without human contact (Nemo) during a) the day (1:00–6:00 PM) with an enlarged portion highlighting the small LF peaks; and b) the evening (6:00–10:00 PM).

In parallel, the power spectra distribution differed among koalas, as well as between the day and the evening, whether considering each period of inactivity successively (Fig. 4) or averaged over the day and night (Fig. 5). Nemo and Nugget's distributions resembled those classically found in the literature [12]–[13] at least during the night. During the day, two weak LF peaks were found in both koalas (one at 0.01 Hz and one at 0.07 Hz), while only one peak subsisted in the evening (the one at 0.01 Hz). HF peaks were found at 0.17–0.23 Hz in both koalas during the evening period but this peak disappeared during the day. The HF range coincided with the respiratory range reported for koalas [16]. In contrast, Crimson's distribution was unusual in that we could not determine LF and HF peaks. Instead, we observed for both day and evening periods, a major peak, which was intermediary in position between the LF and HF peaks of Nemo and Nugget.

**Figure 6:**
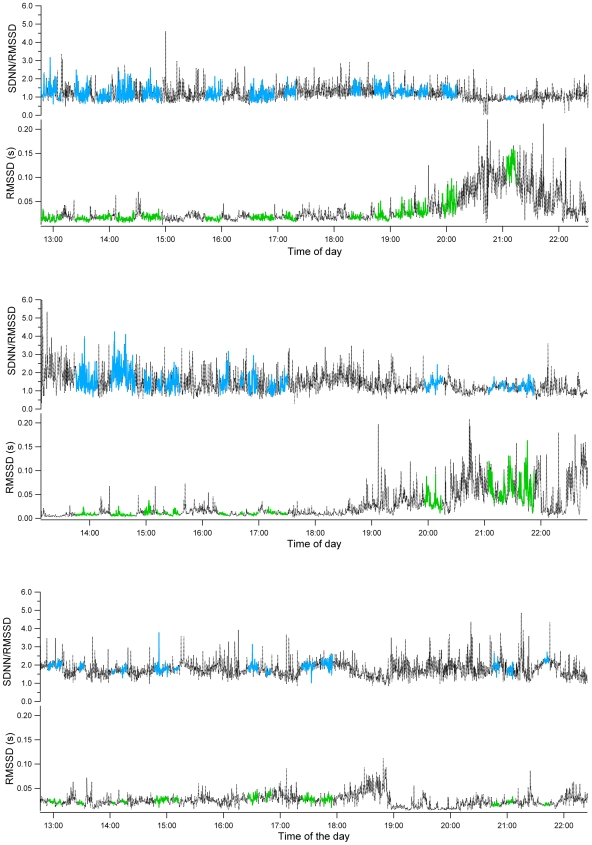
RMSSD and SDNN/RMSSD ratio (see Methods) for a) Nemo, b) Nugget and c) Crimson. In each figure, the lower and upper curves depict the evolution of the RMSSD and the SDNN/RMSSD, respectively, over the complete (dotted black) recording period. Each period of inactivity (green for RMSSD and blue for the ratio) used to calculate the power spectrum of Fig. 4 and 5, are also indicated for information.

The RMSSD and the SDNN/RMSSD evolution over the complete recording session confirmed the results of the power spectrum analysis (Fig. 6). For all koalas, RMSSD values were low during the day and increased at night (F_5,6974_ = 576.7, P<0.0001), while the ratio SDNN/RMSSD became lower at night than during the day for Nemo and Nugget but not for Crimson (F_5,6942_ = 456.5, P<0.0001).

## Discussion

Our study represents, to the best of our knowledge, the first documentation of the continuous evolution of the heart rates of freely-living koalas, over several hours of the day and the evening. The scaling law for marsupials [18], relating the resting heart rate HR to the body mass (M, in kg) as 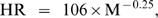
predicts HR values of 63–68 bpm for koalas ranging between 6 and 8 kg (as in our study), which is much lower than the 87 bpm found by [18] for a 6.84 kg captive koala of unknown sex. Note that [18] suspected that their koala might not have been in real resting conditions so that their scaling law may not be a good representation for this species. In addition, the only other report of HR in the literature gives values of 65–90 bpm [19]. In comparison, we measured much higher average heart rate values in our semi-captive koalas during periods of immobility. This is to be expected as our measurements do not represent absolute resting heart rates: we may have been out of the thermoneutrality zone of the animal, they may have eaten prior to the recording session or may have been affected by the presence of tourists (see below).

Moreover, none of the aforementioned studies provide any information about the temporal evolution of koalas' heart rates. Here, we found evidence for a circadian rhythm in cardiac activity of koalas, highlighted by a synchronized change among individuals in the average heart rate values and its variability occurring at the end of the afternoon. This change is to be related with the pattern of activity of koalas that are mostly inactive during the day and show peak of activities, mainly feeding, classically between 5 PM and 12 AM [1]. Interestingly, and apart from Crimson (see below), the evening heart rates were on average lower than those during the day, although bouts of activity led to a transitory tachycardia (but also see below).

We could not find any explanatory variables for this unexpected daily trend, but its synchronicity suggests that it could be driven by an internal clock under the influence of light levels or temperature.

### Crimson's case

If the heart rate of Crimson was clearly higher than that of Nemo (and both Nemo and Nugget in the evening period), its important variability, especially during the day when compared to that of the two other koalas, is cause for concern. We do not believe that Crimson's cardiac response could be due to a problem with the placement of the electrodes since we could record clear ECG peaks. Crimson apparently displayed an elevated heart rate, coupled with an unusual autonomic signal. The single predominant peak of Crimson may be a reflection of parasympathetic activity since it was located in a zone close – yet not similar – to that of the high frequency peak in the power spectral analysis of the other koalas. Interestingly, Crimson's body mass (6 kg) is small for a 2-yrs old koala. In comparison, Nemo's and Nugget's stand at 7 and 8 kg at 2 and 5 years old, respectively. Similarly, [20] reported body masses of 7.0–8.7 kg for a range of unknown age female koalas. Crimson's higher heart rate could be partly explained by her small size, which could be potentially associated to a slightly poorer condition (especially since her heart rate variability is high throughout the observation period). Although the health check did not reveal any specific pathology, we cannot ascertain that she does not suffer from a light cardiac malformation, which could explain her altered cardiac signal. It is important to note that Crimson is affected by consanguinity (her parents were brother and sister). In the light of our results, it appears important to evaluate the potential link between her heart rate abnormality and the situation under which she lives, i.e. whether the presence of tourists may aggravate her condition or not.

### Heart rate and autonomic function

Excluding Crimson, SDNN/RMSSD ratio, which represents the balance between sympathetic and parasympathetic nervous activities, was high during the day and low during the night. In contrast, the RMSSD, which represents the parasympathetic nervous activity, was low during the day and high during the night. This suggests that sympathetic nervous activity was predominantly active during the day while parasympathetic nervous activity was active during the night. It should be noted that the power spectral signals during the day were extremely weak. While parasympathetic dominancy allows vertebrates to modulate rapidly their heart rate [7], [13]–[14], a preponderance of sympathetic nervous activity can be associated with activity or with a stressful situation. Indeed, in the latter case, a corticotrophin-releasing factor is known to increase blood pressure and heart rate by stimulating noradrenergic sympathetic nervous outflow in stressed animals [21]. As koalas were relatively not active during the day, sympathetic dominant activity suggested they were subjected to stress.

In the evening, heart rate variability and both LF and HF peak increased, indicating that parasympathetic activity has increased. As mentioned above, an increase in activity is expected to lead to an increase in sympathetic activity and thus we would expect sympathetic dominant signals in the evening. Yet, although the three koalas displayed more activity during the evening than during the day, this was still limited to a few vertical movements. In addition, feeding and digestion were preponderant and this may explain the observed increase in parasympathetic activity [22].

#### Proximity with tourists

While both Nemo and Nugget displayed weak sympathetic-dominant activity during the day, the exposure to tourists corresponded with Nugget's having a higher HR than that of Nemo. This difference disappeared completely in the evening when both koalas displayed lower HR with higher variability. Whether this difference in heart rate is solely the result of human presence can be debated yet its disappearance in the absence of the stimulus calls for future investigations in this direction. In order to ascertain that the presence of tourists increases the average HR, we would need to conduct intra-individual comparisons, i.e. to compare the HR of Nugget during periods of exposure and non exposure. In the present situation, we were unable to obtain Nugget's HR in the absence of tourists since visitors succeeded to each other within a minimum of two minutes. It is finally interesting to note that walking just under the tree of a koala, as well as the sound of branches cracking, lead to an acute – yet transitory – stress, as this mimics the presence of ground-based predators.

Although based on the minimum sample size possible – an unavoidable limitation when working with vulnerable, captive animals – our results augur for the presence of tourists to have a non-negligible effect on the metabolic activity of koalas despite them showing no exterior signs of weariness. It would be interesting to monitor the intake rates of individuals in the display area and compare it with that in tourist-free areas. We suspect that koala in exhibition area would feed more to compensate for the energy expended in this physiological response, although this can be masked by adaptative processes of the digestive function of female koalas to meet the demands of reproduction [23]. Similarly, measurement of stress hormone levels, such as cortisol [24], would help quantifying the stress imposed by tourist proximity. In conclusion, and in order to confirm our preliminary results, we would like to encourage similar approach to be carried out onto larger samples of koalas and other species in conservation centers.
